# Mass Spectrometry-Based Approaches Toward Absolute Quantitative Proteomics

**DOI:** 10.2174/138920208784533647

**Published:** 2008-06

**Authors:** Keiji Kito, Takashi Ito

**Affiliations:** Department of Computational Biology, Graduate School of Frontier Sciences, University of Tokyo, Kashiwa 277-8561, Japan

**Keywords:** Quantitative proteomics, mass spectrometry, absolute quantification, stable isotope labeling, label-free.

## Abstract

Mass spectrometry has served as a major tool for the discipline of proteomics to catalogue proteins in an unprecedented scale. With chemical and metabolic techniques for stable isotope labeling developed over the past decade, it is now routinely used as a method for relative quantification to provide valuable information on alteration of protein abundance in a proteome-wide scale. More recently, absolute or stoichiometric quantification of proteome is becoming feasible, in particular, with the development of strategies with isotope-labeled standards composed of concatenated peptides. On the other hand, remarkable progress has been also made in label-free quantification methods based on the number of identified peptides. Here we review these mass spectrometry-based approaches for absolute quantification of proteome and discuss their implications.

## 1. INTRODUCTION

Proteomics is a promising discipline in the aim of profiling of whole proteins, identifying their modifications and interactions, and providing abundance information. In so doing, it contributes to reveal molecular systems underlying various biological phenomena and provide valuable information on disease mechanisms as well as biomarkers for diagnosis and prognosis. Mass spectrometry (MS) enables protein identification and quantification in a large-scale, and hence serves as the most powerful tool to address these purposes of proteomics [1-5]. 

In general, proteins are digested with specific proteases, such as trypsin, into a distinct set of peptides. These peptides are then ionized and introduced into MS instruments. Tandem mass spectrometry (MS/MS) has been widely used in proteome analysis, where a peptide ion to be analyzed is selectively isolated and fragmented to obtain an MS/MS spectrum. Since the pattern of these fragment ions is dependent on the amino acid sequence of its precursor ion, it can be compared with theoretical ones calculated from protein sequences in the database. If the MS/MS spectrum measured for a peptide shows a reliable matching with one of the theoretical spectra, the peptide is identified as the one used for the calculation of the theoretical spectrum.

Since the MS/MS analysis identifies individual peptides, it is amenable to be combined with liquid chromatography (LC) as a peptide separation step that precedes MS. LC–MS/MS is a commonly used technical platform, where peptides are separated with reverse-phase LC, directly ionized *via *electrospray ionization, and introduced into mass spectro-meter. Alternatively, peptides eluted from LC are separately collected and spotted on a plate for matrix assisted laser desorption ionization (MALDI). These systems especially have an impact on the analysis of highly complex samples composed of a great number of proteins. Their power can be further enhanced by the use of multidimensional separation with cation-exchange and reverse-phase LC to identify more than 1,000 proteins at once [6-8]. Thus, MS is a high throughput and powerful protein identification system. 

MS has also an ability to provide quantitative information in proteome analysis. Various strategies have been developed to detect relative changes in protein abundance between the samples to be compared [9,  10]. They can be divided into two categories; one is based on stable isotope labeling and the other is the so-called label-free method. 

In the former, samples to be analyzed for relative quantification are differentially labeled with stable isotope, combined, and simultaneously subjected to MS. Ratio of peak intensity between the ions of an isotope pair (i.e., light and heavy peptide ions) gives relative difference in abundance of the protein from which the peptide is derived. Various methods have been developed for stable isotope labeling of proteome, including chemical, proteolytic, and metabolic labeling ones [11-15]. 

By contrast, in the latter or label-free methods, samples to be compared are individually introduced into mass spectrometer, and peak intensities or frequencies of identification measured in separate runs are compared to calculate relative change in protein abundance [9,  10]. Accordingly, label-free strategies are much simpler but more error-prone than isotope labeling ones, due to systematic variations among individual runs and stochastic nature of the indices used for calculation.

Although these techniques are widely used for relative quantification in proteomics studies, the ultimate goal of quantitative proteomics is definitely the absolute measurement of protein abundance. Absolute quantification provides a far more precise description of molecular events in the biological processes than relative quantification. Furthermore, absolute quantification data can be readily exchanged among different studies to facilitate data integration. Recent advent in proteomics has enabled MS-based absolute quantification by extending the technologies originally developed for relative quantification. Thus, we review both relative and absolute quantification techniques either with or without stable isotope labeling.

## 2. QUANTITATIVE TECHNIQUES BASED ON STABLE ISOTOPE LABELING

In stable isotope labeling methods, quantitative values were calculated based on ratio of peak intensity between isotope pair ions, one of which contains only native isotopes whereas the other bears a number of heavy stable isotopes. Having the same chemical properties, two peptide ions of an isotope pair can be simultaneously introduced into mass spectrometer but clearly distinguished by their mass difference. Simultaneous measurement of ion intensities in the same analysis eliminates not only run-to-run variations in performance of LC and MS, amounts of injected sample, and ion-suppression effect of co-eluting ions, but also limitations in intrinsic dynamic range of each MS, thereby enabling more accurate and reliable quantification.

### 2.1. Relative Quantification

In relative quantification, samples to be compared are differentially labeled with stable isotopes. These samples are then combined and subjected to quantitative MS. Peak intensity ratio between heavy and light peptides is measured to learn relative change in protein abundance. Various labeling methods have been developed, including chemical, proteolytic, isobaric, and metabolic labeling techniques.

The most popular method of chemical labeling would be the isotope-coded affinity tags (ICAT) approach, in which a compound containing stable isotope is coupled to Cys residues in proteins [16,  17]. Different isotopomers of the compound, each having a unique mass, are used for labeling of different samples. Following this differential labeling procedure, the samples are mixed and subjected to protease digestion followed by affinity-purification of Cys-containing peptides. Besides the original ICAT methods, strategies have been reported for chemical labeling of carboxyl, amino, or thiol moieties [11-15,  18]. In the labeling method coupled with hydrolysis, proteins are digested with protease in the presence of ^18^O-labeled water so that ^18^O is incorporated at the carboxyl end of each peptide [19,  20]. Another *in vitro* labeling method is an isobaric tagging strategy, in which each tag has an identical mass but contains stable isotopes at unique atomic positions to generate a reporter ion with a unique mass-charge ratio upon fragmentation [21,  22]. 

An obvious advantage of these *in vitro* labeling methods is that they can be applied to tissue samples, for which *in vivo* labeling is difficult or practically impossible. On the other hand, they require tangled procedures for sample handling and labeling. Accordingly, the samples to be compared are forced to be combined at later stages of the procedure, allowing variations in earlier steps to affect accuracy of quantification.

An alternative labeling method is the *in vivo* metabolic incorporation of stable isotopes, where cells are cultivated in a medium supplemented with an appropriate stable isotope-labeled nutrient that is essential for growth to achieve labeling of whole proteome [23-26]. Amino acids are usually used as the labeled essential nutrient, and such a procedure is often called SILAC for stable isotope labeling by amino acids in cell culture [24]. Proteome from multicellular and mammalian organisms, such as worm, fly, and rat, can be also metabolically labeled by feeding stable isotope-labeled microorganisms [27,  28]. 

An obvious advantage of these metabolic labeling methods over the chemical and hydrolytic ones is that protein samples can be combined at much earlier step in the procedure, for instance, at the stage of cell harvest. Accordingly, the effect of experimental errors can be minimized. On the other hand, it is difficult, or even impossible, to complete stable isotope labeling of animal tissues and metabolically inactive cells. To partly circumvent these difficulties, an interesting method termed culture-derived isotope tags (CDITs) was developed, in which relative abundance of proteins in the tissues refractory to metabolic labeling are quantified using the isotope-labeled proteome of a cell line derived from the tissue [29].

These methods have been widely implemented in relative quantification in proteomics studies. With the remarkable analytical power of multidimensional LC, relative differences of several hundred proteins were successfully quantified among yeast cells grown in different culture conditions [30-33]. The strategy was also applied to distinguish contaminants in purification; quantitative comparison between purified and mock-purified samples revealed specific components in a protein complex and a cellular compartment [34,  35]. It can be combined with specific purification techniques for protein complex and post-translationally modified peptides to grasp dynamics of protein interactions [34,  36-40] and phosphorylation in stimulated cells [38,  41-45].

### 2.2. Absolute Quantification

**Fig. (1). Strategies for absolute quantification using stable isotope-labeled standards.**
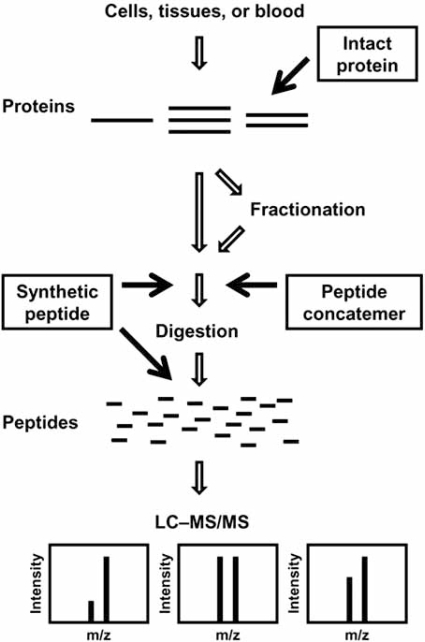
Different types of stable isotope standard are spiked at different steps of the sample preparation procedure. Intact protein standard can be spiked as soon as proteins are extracted from cells, tissues or bloods, even if subsequent fractionation steps (e.g., SDS-PAGE, gel filtration) are included in the procedure. While synthetic peptide standard is spiked before or after digestion with protease, peptide-concatenated standard has to be spiked prior to digestion to allow co-proteolysis of target and standard. Note that synthetic peptide and peptide concatenated standard have to be spiked after protein fractionation steps.

In MS-based absolute quantification, a known amount of isotope-labeled authentic standard is mixed with the analyte, and the mixture is introduced into mass spectrometer. The absolute amount of the analyte is calculated from the ratio of ion intensity between the analyte and its standard. Accordingly, known amounts of stable isotope-labeled synthetic peptides, proteins, or peptide concatemers have been used as a standard for absolute or stoichiometric quantification of proteins. Different types of standard are added to the samples at the different stages of the procedure, and have distinct pros and cons (Fig. **1** and Table **1**). Accordingly, the most suitable standard should be selected, depending on the purpose of the experiment, or on whether it intends to quantify a small number of targets including their post-translational modifications, obtain highly accurate data for a single unique protein, or measure absolute or stoichiometric abundance of many proteins.

#### 2.2.1. Quantification with Stable Isotope-Labeled Peptide Standard

In the original report that uses a stable isotope-labeled peptide as an internal standard for MS-based absolute quantification [46], the amount of endogenous enkephalin in thalamus extract was measured with an ^18^O-incorporated standard peptide. To reduce background noises and enhance the specificity of measurement, multiple reaction monitoring (MRM) mode, in which intensity of multiple fragment ions but not the peptide ion are monitored, was used for absolute quantification of enkaphalin in human tissue [47].

Stable isotope-labeled peptides were initially applied to absolute quantification of a specific protein [48]; three peptides from apolipoprotein A-I were selected, ^2^H- and ^13^C-labeled, and used as standard for quantification of its abundance in human serum. To increase specificity and sensitivity, measurement of difference in intensities of fragment ion(s) [SRM (selected reaction monitoring) or MRM mode] between analyte and standard peptide was implemented in absolute quantification of protein. Expression level of G protein-coupled receptor rhodopsin in rod outer segment membrane was quantified using a ^2^H-labeled synthetic peptide [49]. For quantification of low abundance proteins, enrichment of the target protein is necessary. For instance, following SDS-PAGE separation of yeast extract, a gel slice containing the target protein was excised, mixed with ^13^C and ^15^N-labeled standard peptides, and subjected to trypsin digestion followed by MS [50]. In this study, abundance of Sir2 and Sir4 were determined as ~10^3^ copies per cell. Absolute abundance of proteins in blood or tissue was also quantified using synthetic peptides as isotope-labeled standards in the MRM mode. For instance, absolute amount of C-reactive protein, a well-known diagnostic marker for rheumatoid arthritis, was measured in human serum depleted of abundant proteins (serum albumin and immunoglobulin G) [51]. Similarly, GST-α in human liver, a marker for acute hepatocyte damage, was quantified distinctly from other isozymes [52].

More recently, stable isotope-labeled synthetic peptides were applied to simultaneous quantification of multiple proteins. For instance, the stoichiometry among the 10 components of human spliceosomal U1 small ribonucleoprotein complex was determined using chemical labeling of sample-derived and synthetic standard peptides with isotope-coded reagents after trypsin digestion [53]. Similarly, concentrations of 8 endogenous proteins in human serum were quantified by spiking isotope-labeled standard peptides [54]. In another study, isotope-labeled peptides were applied to absolute quantification of 32 key proteins in the postsynaptic density of rat, including calmodulin-dependent protein kinase, synaptic GTPase-activating protein, glutamate receptors, and scaffold proteins [55]; the absolute and stoichiometric abundance obtained from this study provided valuable information on abundance of receptor subtypes and protein interactions. 

Taken together, isotope-labeled synthetic peptide is definitely a powerful tool for absolute quantification not only for cultured cells, but also for tissue or blood samples from animals and human. However, it is too expensive to synthesize multiple stable isotope-labeled peptides for quantification of multiple proteins. In this context, it is interesting to note that the CDIT strategy described above allows quantification of tissue proteins without any labeled synthetic peptides as follows [29]. Absolute amounts of the proteins in the cultured cell line labeled *in vivo* are determined with unlabeled synthetic peptides. These quantified labeled proteins are, in turn, used as the standards for absolute quantification of unlabeled tissue proteins. Indeed, this strategy was successfully used to quantify103 proteins in the mouse brain.

Peptide standard can be used to quantify not only protein abundance but also post-translational modifications. Isotope-labeled unphosphorylated and phosphorylated peptides can be used to quantify phosphorylation stoichiometry. For instance, stoichiometry of phosphorylation at Ser-1126 of separase, which negatively regulates its activity, was measured in different stages of the cell cycle either in a peptide ion scanning mode [56] or in SRM mode [50]. Simultaneous quantification of multiple phosphoryation sites was also reported. Two phosphorylated sites of cyclin-dependent kinases, which inhibit their activity, are regulated in a cell cycle-specific manner. Stoichiometry of four possible patterns of these phosphorylation sites was determined to reveal that isoforms unphosphorylated and phosphorylated at both sites were dominant in M-phase and S-phase, respectively, whereas those phosphorylated at either site were minorities [57]. 

Modifications other than phosphorylation can be quantified. For instance, abundance of polyubiquitin chain branched at the Lys-48 was measured in mammalian cells treated with or without the proteasome inhibitor MG132 [58]. Ubiquitination is mediated *via *an isopeptide bond between the carboxy-terminal end of ubiquitin and the ε-amino group of a Lys residue in the substrate. Since the amino acid sequence at the C-terminal end of ubiquitin is Arg-Gly-Gly, tryptic digestion of ubiquitinated proteins produces peptides containing Lys conjugated with Gly-Gly or diglycine at its ε-amino group. In a study aiming at absolute quantification of ubiquitin conjugated sites, a peptide bearing diglycine-conjugated Lys residue was synthesized and used as an isotope-labeled standard. Topology of polyubiquitin chain of an *in vitro* ubiquitinated protein, or which of the seven Lys residues is used for branching, was also analyzed using ubiquitin-standard peptides [59]. Beside phosphorylation and ubiquitination, absolute quantification of farnesylation was reported for H-Ras using a ^2^H-labeled, farnesylated carboxy-terminal peptide [60].

#### 2.2.2. Quantification with Stable Isotope-Labeled Intact Protein

When a proteolytic standard peptide is used for absolute quantification, efficiency of protease digestion is critically important for accuracy, because incomplete digestion of the analyte leads to underestime its amount. While some studies optimized and monitored cleavage efficiency for specific peptides [48,  49], it is difficult to assess a large number of peptides in terms of cleavage efficiency. Notably, measured abundances of an analyte protein can differ substantially, depending on the tryptic peptides used as the standard, presumably because efficiency of trypsin digestion is different from one site to another [51]. In particular, when SDS-PAGE is used as a pre-fractionation step, recovery of peptides is affected not only by the efficiency of in-gel digestion but also by that of peptide extraction from gel slices [61].

An ideal standard for absolute quantification of a protein is obviously the protein *per se* that is labeled with stable isotope, because it can be spiked at the earliest stage of sample preparation to minimize experimental errors and shares exactly the same efficiency of protease digestion with the target proteins in the sample. For instance, concentrations of insulin in sera of normal individuals and diabetic patients were quantified with an isotope-labeled recombinant standard protein expressed in and purified from *E. coli* [62]. Similarly, ^15^N-labeled recombinant standard proteins were used for absolute quantification of 6 proteins localized in postsynaptic density [63], and expression level of alcohol dehydrogenase isozyme ADH1C1 was quantified in human liver tissue using a ^13^C- and ^15^N-labeled recombinant intact protein [64]. 

An intriguing study was reported to directly compare two strategies, one with a synthetic peptide standard and the other with an intact protein standard, in absolute quantification of *Staphylococcus aureus* superantigenic toxins spiked into drinking water and urine samples [65]. The amount quantified with the synthetic peptide standards was smaller than that obtained with the intact protein standard, presumably because of incomplete digestion of the toxins in sample. This result indicates the power of the strategy using an intact protein as a stable isotope standard.

#### 2.2.3. Quantification with Stable Isotope-Labeled Peptide-Concatenated Standard

Although the strategies using synthetic peptides or intact proteins as stable isotope-labeled standards allow us to learn absolute amounts of specific proteins, a large scale analysis requires preparation and handling of many standard peptides/proteins, thereby raising many concerns. First, it is expensive to prepare many stable isotope-labeled synthetic peptides. Second, the purity of synthetic peptides is variable from one to another and is often unsatisfactory for accurate quantification. Third, it is also a daunting task to express and purify many recombinant proteins as stable isotope-labeled standards. Forth, since individual standards are differentially lost during the course of experiments, one cannot guarantee their precise amounts or even their equimolarity.

**Fig. (2). Stable isotope-labeled and peptide-concatenated standard for absolute or stoichiometric quantification.**
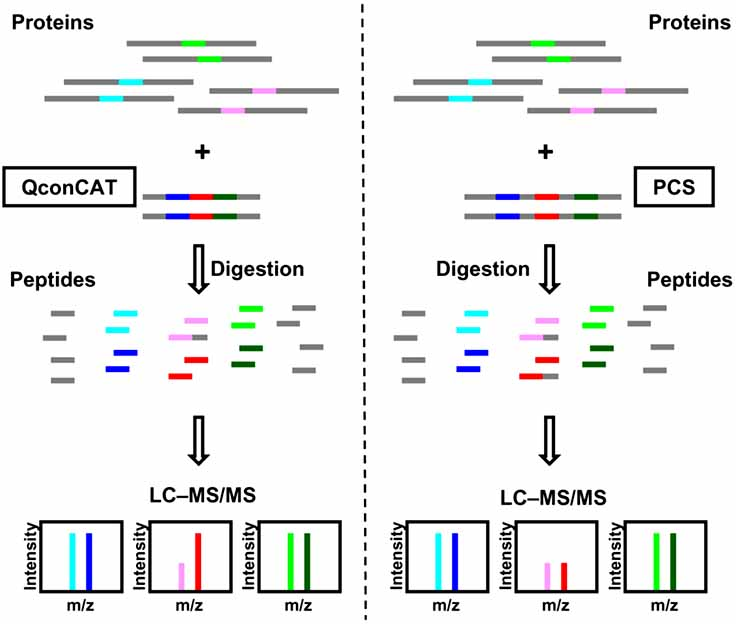
In strategy using peptide-concatenated standard, tryptic peptides to be quantified are selected from target proteins and concatenated to generate an artificial protein, which serves as a stable isotope-labeled standard. Colored fragments denote the selected tryptic peptides. Target and standard peptides that form an isotope pair are indicated by similar colors (blue and light blue, red and pink, and green and light green). Peptide-concatenation not only guarantees equimolar spiking of every standard peptide but also eliminates the need to prepare many standard molecules. Two types of peptide-concatenated standard, namely QconCAT [66] and PCS [67], have been reported. While each peptide included in QconCAT is the tryptic peptide per se, that in PCS carries its natural flanking sequences at both sides to faithfully recapitulate the efficiency of proteolytic cleavage in the target protein. Even a peptide excised less efficiently from the target protein (colored in pink) can be precisely quantified with PCS, because its standard (colored in red) is also excised from the PCS at a similar efficiency.

To overcome these bottlenecks, two groups independently conceived a strategy that uses a peptide concatemer as a standard, namely QconCAT [66] and PCS for peptide-concatenated standard [67]. In both strategies, tryptic peptides used for quantification are concatenated into a single artificial protein. This protein was metabolically labeled with stable isotope in *E. coli*, purified, and mixed with a protein sample to obtain absolute or stoichiometric quantities of multiple proteins (Fig. **2**). This peptide concatenation strategy eliminates the daunting task for the preparation of many standard peptides/proteins. Furthermore, since all isotope-labeled peptides are contained in a single protein, they are always added to the sample at exactly the same molarity. The QconCAT approach provided absolute quantitative data of more than 10 proteins in chick skeletal muscle of different developmental stages [66,  68]. It was also used for quantification of plasma proteins [69]; absolute amounts of 13 proteins in human plasma of 20–10,000 fmol/μl concentrations were successfully measured in an MRM mode.

However, it should be noted that, as discussed above, one of the critical keys for accurate quantification is to equalize cleavage efficiency between the analyte and its standard. It was shown that the amino acid context around a trypsin cleavage site substantially affects the efficiency of its digestion [70,  71]. In contrast to QconCAT, PCS contains each standard peptide with its natural flanking sequences on both sides to faithfully recapitulate the efficiency of tryptic cleavage of parental proteins or analytes (Fig. **2**). The involvement of flanking sequences was demonstrated to improve the accuracy of quantification, and led to accurate quantification of stoichiometry among 5 subunits in eIF2B stable complex of yeast within 5% measurement error [67]. Similarly, others successfully quantified absolute and stoichiometric abundance of each subunit of transducin, a heterotrimeric G-protein, using a PCS incorporating the flanking sequences of each tryptic peptide [72]. Notably, the QconCAT strategy, which lacks flanking sequences, resulted in an underestimation of the amounts of *Staphylococcus* toxins spiked into drinking water and urine samples when compared to the strategy using an intact protein standard [65], presumably because the target protein was less efficiently digested than the QconCAT. These studies highlight the importance of incorporation of flanking sequences into the standard to ensure highly accurate absolute or stoichiometric quantification.

Peptide-concatenated artificial proteins are, in most cases, recovered in insoluble fraction. Since the order of the peptides affect the success rate of production in an *in vitro* translation system [73], an efficient algorithm is needed to optimize the peptide order for improvement of the solubility of peptide-concatenated standard proteins. 

The insoluble-prone nature of peptide-concatenated artificial proteins not only makes their handling difficult but also limits their sizes. Thus, many standards have to be used concurrently in a large scale analysis. It then becomes important to know precise amounts of the standards to integrate quantitative data obtained from each of them. To solve this issue and to expand the scale of analysis, we proposed a hierarchical PCS strategy, in which each primary PCS includes a unique “bar-code” peptide and is quantified by a secondary PCS composed of the bar-code peptides [67]. It is also possible to adjust the amount of each primary PCS to extend the dynamic range of quantification. 

## 3. QUANTITATIVE TECHNIQUES BASED ON LABEL-FREE STRATEGIES

**Fig. (3). Label-free methods for absolute quantitative proteomics.**
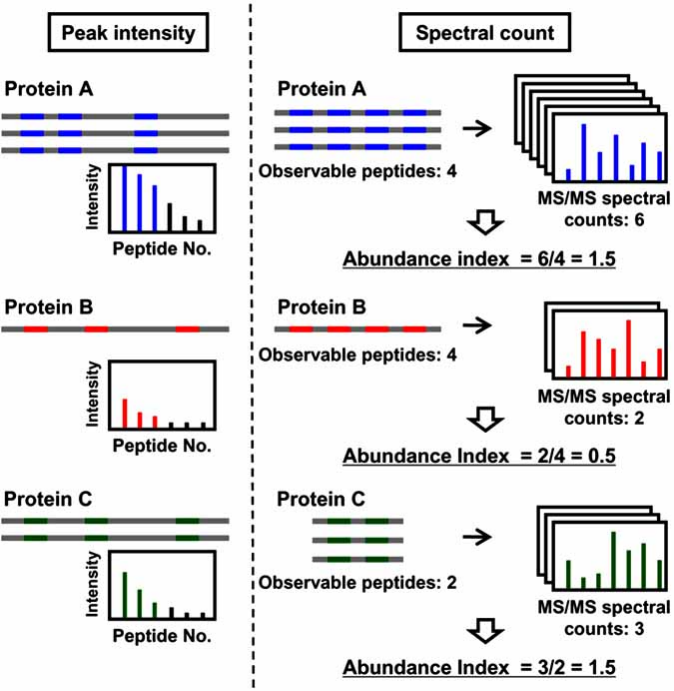
In the strategy based on intensities of individual peptides, average of intensities of the three most intense ions is used to generate reliable absolute quantitative data. In the strategy based on identification frequency, spectral count (i.e., number of identified MS/MS spectra) is the most useful indicator to estimate protein abundance. Since larger proteins tend to provide more observable peptides than smaller ones, each spectral count is divided by observable peptide number for normalization to ensure accuracy of quantification. For instance, while proteins A and C present at the same abundance have different spectral counts (i.e., 6 and 3, respectively), they share the same normalized spectral count (i.e., 1.5).

Stable isotope-labeling strategies described above have enabled quantitative MS-based proteomics. However, they inevitably require additional steps for isotope labeling and/or preparation of the standards. By contrast, the so-called label-free (standard-free) method is simple and requires no additional experimental steps; it just exploits peak intensity of peptide ion or identification frequency for a particular protein to obtain quantitative data (Fig. **3** and Table **1**). While absolute quantification with stable-isotope labeling can quantify only the proteins with corresponding isotope standards, label-free strategies can, in principle, quantify any protein from which a peptide is unambiguously identified. This implies that the label-free methods are amenable to a large scale analysis. On the other hand, they provide less accurate quantitative values than those by label-based ones, due to run-to-run variations and a stochastic nature of the measurement.

### 3.1. Relative Quantification

#### 3.1.1. Quantification Based on Peak Intensity

In relative quantification based on peak intensity, each sample is separately subjected to MS. Peptide peak intensity is measured in individual runs and change in protein abundance is calculated *via *a comparison among different analyses. This approach has been applied to quantification of relative change in protein expression [74,  75] and quantitative profiling of purified proteins to identify *bona fide* components of a protein complex and reveal dynamics of protein-protein interactions [76,  77].

In contrast to stable isotope labeling methods, label-free approach based on peak intensity is error-prone [78], due to run-to-run variations in performance of LC and MS, amounts of injected samples, and ion-suppression effect of co-detected ions, and also due to limited dynamic range of each mass spectrometry. Systematic errors induced by the first two factors can be normalized by spiking an identical amount of standard protein into every sample to be compared [79,  80], the total ion intensities over the entire analysis [74], or using the average intensity ratios between target peptide and co-eluting peptides as a pseudo internal standard [81]. Furthermore, high reproducibility of retention time for each peptide is required to extract an ion pair from different runs, when either, but not both, of the pair is identified in MS/MS analysis. Otherwise, an algorithm to align peptide ion maps of different analyses has to be developed to compare peak intensity of each peptide ion [82].

#### 3.1.2. Quantitative Approaches Based on Identification Frequency

An increase in protein abundance usually results in an increase in the number of identifications of its tryptic peptides, and *vice versa*. Thus, identification frequency, which is the number of identified peptides, precursor ions, or MS/MS spectra (spectral count) for each protein, can be used to estimate relative difference in protein abundance. Peptide identification number was originally applied to quantitative proteomics analysis of urine sample from healthy donors and patients [83]. Among the factors of identification frequencies, spectral count showed the highest correlation with relative protein abundance, suggesting it to be the best index for relative quantification [8]. Relative quantitative approaches were also taken to compare protein expression in yeast and mammalian cells under different culture conditions [74,  84,  85], and the screening of phosphotyrosine-binding proteins in mammalian cells [86].

An intriguing study compared relative abundance calculated from the spectral count with that obtained using a stable isotope-labeling method [85]. When only the peptides with high signal-to-noise ratio in the extracted ion chromatogram were included in the calculation, the two methods showed a positive correlation within 1.5-fold error. Quantitative data determined by isotope labeling and spectral count would compensate each other to improve accuracy of quantification.

### 3.2. Absolute Quantification

#### 3.2.1. Quantification Based on Peak Intensity

Although difference in peak intensity has been exploited for relative quantification, individual peptides differ in propensities to be ionized and in efficiencies for isolation and detection, and they are also dependent on MS equipments. Accordingly, ion intensities can be different even among the peptides present at the same molarity (e.g., peptides derived from a single protein). Thus, these differences should be corrected for absolute quantification.

To reduce the effect of variance in individual peak intensities, average of ion intensities for multiple peptides in a particular protein was used as a quantitative value to estimate protein abundance [87]. In this study, a relationship was observed between protein abundance and average of intensities of the three most intense peptide ions. The average for the three most intense ions had an ability to predict the abundance of known amount of proteins with less than ~15% error. Stoichiometry of GroEL and GroES of *E.coli* was successfully quantified to be 2:1, consistent with known structure of this molecular chaperone. While proven useful by an evaluation using a mixture of known amounts of proteins, this strategy may generate a large error for low abundance proteins, for which only a small number of peptides can be identified, and fails to quantify proteins from which only one or two peptides are identified.

#### 3.2.2. Quantitative Approaches Based on Identification Frequency

Label-free approaches based on identification frequency, which had been applied to relative quantification, was also modified to estimate absolute protein abundance. Larger proteins have more peptides that are detectable by MS than smaller ones. Accordingly, the number of identified peptides, precursor ions, and MS/MS spectra (spectra count) may be different between the two proteins that exist at the same abundance but differ in sizes. Thus, to know absolute or stoichiometric quantity, one should use the percentile fraction of the protein sequence covered by identified peptides or normalize the number of identifications by either protein size or observable peptide kinds.

##### 3.2.2.1. Normalization with Protein Size

It was observed that the number of identified peptides correlates with the codon adaptaion index of the protein, which serves as an indicator of protein abundance [6]. The number of identified peptides per protein molecular weight provided stoichiometric abundance for clathrin and its adaptor proteins in clathrin-coated vesicles [88]. Clustering analysis using abundance index, or the number of identified precursor ions per protein molecular weight, successfully identified proteins associated with SAGA, a histone acetyltransferase complex, as those having a similar pattern of purification abundance [89]. Spectral count per protein length was further optimized to give normalized spectral abundance factor (NSAF); the latter index is calculated by dividing the former by the sum of all spectral count per protein length in each MS run to eliminate variation of each analysis [90]. Quantitative analysis with NSAF revealed the subunit stoichiometry of yeast Mediator, a transcriptional coactivator complex [91]. A study examined correlations between relative protein abundance and three indices (i.e., sequence coverage, identified peptide number, and spectral count) by spiking known amounts of 6 different proteins into yeast cell extract [8]. While sequence coverage and peptide numbers failed to linearly correlate with relative abundance, a strong correlation was observed between relative abundance and spectral count with 2-order of magnitude. Furthermore, spectral count per molecular weight of each protein had a linear correlation with stoichiometry of 6 different proteins [8], suggesting that spectral count is the most useful index for absolute or stoichiometric quantification based on the frequency of identifications.

##### 3.2.2.2. Normalization with Observable Peptides

The number of observable peptides in each protein has been used as an alternative normalization factor. For instance, the protein abundance index (PAI) is calculated by dividing the number of identified precursor ions by the number of theoretically observable tryptic peptides for each protein, to roughly estimate protein abundance [92]. This index was later refined to be emPAI or exponentially modified PAI (i.e., 10^PAI^–1), where theoretically observable peptides were defined as those within a range of mass-to-charge ratio of scanning in mass spectrometer [93]. The emPAI demonstrated its ability by successfully estimating absolute abundance of 46 proteins, which had been measured using synthetic peptides, with 2–3 of average deviation factor: more than 2- to 3-fold difference in absolute abundance can be detected with this index. The values of emPAI can be calculated so easily that it is quite useful in obtaining an approximation of absolute protein abundance in a large-scale analysis.

Sequence coverage rate and the identification frequency per protein length or theoretically observable tryptic peptide kinds have a correlation with protein abundance and hence serve as useful indices to obtain absolute quantification data. Meanwhile, individual peptides have different propensities to be detected and identified by MS/MS analysis, not only because they differ in efficiency of proteolytic digestion, ionization, and detection in mass spectrometer, but because qualities and patterns of fragment ions are variable depending on their amino acid composition.

To sophisticate the approaches based on peptide and spectral count, observability of each peptide, or a probability that the peptide is identified with MS analysis, was recently introduced as a novel index [94,  95]. Absolute protein expression profiling approach, termed APEX, was developed where peptide observability was predicted from 4,023 tryptic peptides of 40 abundant proteins identified in a shotgun analysis of the yeast proteome [95]. Using this dataset containing 714 observed and 3,309 not-observed peptides, a probability for identification of each peptide from the yeast proteome was calculated based on the frequencies of each amino acid, peptide length, and molecular weight. Redundant spectral count was normalized by the sum of observability for each peptide from a corresponding protein and by the probability of protein identification calculated by ProteinProphet [96]. Resultant values were divided by the sum of the values of all identified proteins to generate an APEX score that would provide an absolute value of protein abundance. APEX successfully measured the abundance of 10 proteins, known amounts of which had been spiked into yeast cell extract, with mean difference of approximately 2-fold at 2-order magnitude: this approach had an ability to detect more than 2-fold difference in protein abundance.

Another group computationally predicted observable peptides, termed ‘proteotypic peptides’, using a much larger dataset composed of more than 600,000 peptides from yeast proteins identified on four different experimental methods [97]. A proteotypic peptide is defined as the peptide that was detected in more than half of the proteomics studies in which the protein was detected. Approximately 500 physiochemical properties of more than 16,000 proteotypic peptides from 4,030 yeast proteins were used to develop a classifier that distinguishes between proteotypic and non-proteotypic peptides. Proteotypic peptides were successfully predicted with 65–80% coverage and less than 10% error. As originally suggested [98], a dataset of proteotypic peptides would be of particular use in selecting peptides to be used as standards in quantification with stable isotope-labeling. It would be also useful to generate a library of selected information-rich peptides for the reduction of time for database search and the improvement of accuracy of identification [99].

Prediction of peptide observability is an important factor for normalization of identified peptide number and spectral count to generate a more accurate index for estimating protein abundance. However, it should be noted that the probability of peptide identification would be dependent on the experimental designs, types of MS instruments, and analytical conditions, as suggested by the studies of prediction for different data sets [100]. Indeed, propensity of a peptide to be proteotypic was shown to depend on experimental methods [97]. Thus, application of classification values generated from a dataset to other datasets may substantially compromise both accuracy of prediction and coverage. Customized score for peptide observability may help generate more accurate estimation of absolute protein abundance. 

## 4. COVERAGE AND DYNAMIC RANGE OF MASS SPECTROMETRY BASED PROTEOMICS

Current potential of MS-based proteomics still falls short of covering entire proteome. Fractionation of protein or peptide mixture prior to MS can improve the coverage of protein identification over proteome. For yeast proteome, protein separation with SDS–PAGE prior to application to LC–MS analysis or peptide fractionations *via *two- or three-dimensional LC led to identification of 1,500–2,000 proteins [6-8,  101,  102]. However, even the coverage of these analyses corresponds to ~30% of the yeast proteome. By contrast, western-blotting approach over yeast proteome, in which each yeast ORF tagged with TAP-tag was immunodetected, provided absolute quantity as copies per cell for 4,251 proteins or ~70% of the total proteome [103]. While MS is a general system for protein identification that does not require any specific strains such as the tagged strains, its current sensitivity in detection of a particular protein in highly complex samples (e.g., total cell extract) is substantially lower than targeted detection systems such as western-blotting. More recently, a high coverage of fly proteome was achieved by combining multiple separation procedures including fractionation of cellular compartments, protein separation using gel filtration and isoelectric focusing, and peptide separation with multidimensional LC [104]. This study succeeded in cataloguing more than 9,000 proteins to cover ~60% of the fly proteome. Furthermore, combination of different types of MS and experimental methods proved to increase the sensitivity of protein identification significantly [105,  106]. All possible means to increase coverage have to be combined to achieve a truly proteome-wide quantitative analysis.

Accurate quantification of absolute abundance requires high specificity and wide dynamic range. Background noise peak and co-detected irrelevant ions often interfere with detection of weak target peaks, thereby affecting both specificity and dynamic range. To reduce background noise and enhance specificity and dynamic range, SRM or MRM data acquisition mode, in which intensity of selected or multiple fragment ions other than peptide ion itself are monitored, have been used for measurement of absolute quantity [47,  49,  51,  55,  64,  69]. Combination of SRM mode and protein separation with SDS-PAGE was shown to allow quantification of low abundance proteins (~10^3^ copies per cell) [50]. Quantification in MRM mode for peptide mixture pre-fractionated with strong cation-exchange LC allowed us to measure absolute amounts of proteins spiked in plasma at the concentration of 1–10 ng/ml [107]. High-resolution MS is an alternative technical basis to increase specificity and dynamic range, because it can better separate an analyte from co-eluting peptides with similar mass-to-charge ratio. High-resolution can also generate spectrum with low background noise leading to an increase in dynamic range. For instance, LTQ-Orbitrap mass spectrometer [108], recently released into proteomics field, has achieved a strong linearity in quantification of spiked proteins within 4-orders of dynamic range [109].

## 5. CONCLUSIONS

The pros and cons of stable isotope-labeling strategies and label-free approaches were summarized in terms of absolute quantification (Table (**1**)). Methods based on stable isotope standard would provide accurate quantitative data, because of calculation *via *the ratio of co-detected ion pair, one from the target and the other from the standard. In these methods, quality of quantified peak (e.g., signal-to-noise ratio) is a critical factor to affect accuracy and dynamic range. By contrast, strategies for absolute quantification based on indices of normalized identification frequency are easy to implement with no additional experimental step and can be applied to a high throughput and comprehensive analysis. However, these methods have an intrinsic limitation in their accuracy, in particular, for low abundance proteins from which only a small number of peptides is identified. Assuming that both strategies would compensate with each other, we can propose a combined strategy where a proteome-wide estimation of abundance are achieved with label-free methods and corrected using a limited number of stable isotope-labeled standard spiked into the sample. Among the strategies using stable isotope labeling, the peptide concatenation, such as PCS, would be the most versatile one to achieve accurate quantification of absolute protein abundance in a large scale. These quantitative techniques along with the advance in detection coverage would eventually lead to absolute quantification over whole proteome, thereby significantly contributing to both basic and applied studies in various fields of biology and medicine.

## Figures and Tables

**Table 1:** Summary of Approaches for Absolute Quantification of Protein Abundance with Mass Spectrometry

	Standard Types	Measured Values	Spiking Time Point	Normalization	Accuracy	Coverage	Applicability to Post-Translational Modification	Noise Origins
Isotope labeling	Synthetic peptide	Ratio to standard	Before or after digestion	—	Medium	Low	Applicable	S/N of ion peak, Missed cleavage
	Intact protein	Ratio to standard	Just after protein extraction	—	Very High	Low	Not	S/N of ion peak
	Peptide concatemer (QconCAT, PCS)	Ratio to standard	Prior to digestion	—	High	Medium	Not	S/N of ion peak, Missed cleavage (QconCAT)
Label-free	—	Peak intensity	—	Average of most intense three peaks	Low	Medium	Not	Variation of ionization efficiency
	—	Spectral count	—	Observable peptides	Low	High	Not	Stochastically calculated index

## References

[1] Pandey A, Mann M (2000). Proteomics to study genes and genomes. Nature.

[2] Aebersold R, Goodlett DR (2001). Mass spectrometry in proteomics. Chem. Rev.

[3] Aebersold R, Mann M (2003). Mass spectrometry-based proteomics. Nature.

[4] Smith JC, Lambert JP, Elisma F, Figeys D (2007). Proteomics in 2005/2006: developments, applications and challenges. Anal. Chem.

[5] Cravatt BF, Simon GM, Yates JR III (2007). The biological impact of mass-spectrometry-based proteomics. Nature.

[6] Washburn MP, Wolters D, Yates JR III (2001). Large-scale analysis of the yeast proteome by multidimensional protein identification technology. Nat. Biotechnol.

[7] Peng J, Elias JE, Thoreen CC, Licklider LJ, Gygi SP (2003). Evaluation of multidimensional chromatography coupled with tandem mass spectrometry (LC/LC-MS/MS) for large-scale protein analysis: the yeast proteome. J. Proteome Res.

[8] Liu H, Sadygov RG, Yates JR III (2004). A model for random sampling and estimation of relative protein abundance in shotgun proteomics. Anal. Chem.

[9] Bantscheff M, Schirle M, Sweetman G, Rick J, Kuster B (2007). Quantitative mass spectrometry in proteomics: a critical review. Anal. Bioanal. Chem.

[10] Mueller LN, Brusniak MY, Mani DR, Aebersold R (2008). An assessment of software solutions for the analysis of mass spectrometry based quantitative proteomics data. J. Proteome Res.

[11] Ong SE, Foster LJ, Mann M (2003). Mass spectrometric-based approaches in quantitative proteomics. Methods.

[12] Sechi S, Oda Y (2003). Quantitative proteomics using mass spectrometry. Curr. Opin. Chem. Biol.

[13] Goshe MB, Smith RD (2003). Stable isotope-coded proteomic mass spectrometry. Curr. Opin. Biotechnol.

[14] Tao WA, Aebersold R (2003). Advances in quantitative proteomics *via* stable isotope tagging and mass spectrometry. Curr. Opin. Biotechnol.

[15] Ong SE, Mann M (2005). Mass spectrometry-based proteomics turns quantitative. Nat. Chem. Biol.

[16] Gygi SP, Rist B, Gerber SA, Turecek F, Gelb MH, Aebersold R (1999). Quantitative analysis of complex protein mixtures using isotope-coded affinity tags. Nat. Biotechnol.

[17] Han DK, Eng J, Zhou H, Aebersold R (2001). Quantitative profiling of differentiation-induced microsomal proteins using isotope-coded affinity tags and mass spectrometry. Nat. Biotechnol.

[18] Regnier FE, Julka S (2006). Primary amine coding as a path to comparative proteomics. Proteomics.

[19] Yao X, Freas A, Ramirez J, Demirev PA, Fenselau C (2001). Proteolytic ^18^O labeling for comparative proteomics: model studies with two serotypes of adenovirus. Anal. Chem.

[20] Stewart II, Thomson T, Figeys D (2001). ^18^O labeling: a tool for proteomics. Rapid Commun. Mass Spectrom.

[21] Thompson A, Schäfer J, Kuhn K, Kienle S, Schwarz J, Schmidt G, Neumann T, Johnstone R, Mohammed AK, Hamon C (2003). Tandem mass tags: a novel quantification strategy for comparative analysis of complex protein mixtures by MS/MS. Anal. Chem.

[22] Ross PL, Huang YN, Marchese JN, Williamson B, Parker K, Hattan S, Khainovski N, Pillai S, Dey S, Daniels S, Purkayastha S, Juhasz P, Martin S, Bartlet-Jones M, He F, Jacobson A, Pappin DJ (2004). Multiplexed protein quantitation in *Saccharomyces cerevisiae* using amine-reactive isobaric tagging reagents. Mol. Cell Proteomics.

[23] Oda Y, Huang K, Cross FR, Cowburn D, Chait BT (1999). Accurate quantitation of protein expression and site-specific phosphorylation. Proc. Natl. Acad. Sci. USA.

[24] Ong SE, Blagoev B, Kratchmarova I, Kristensen DB, Steen H, Pandey A, Mann M (2002). Stable isotope labeling by amino acids in cell culture, SILAC, as a simple and accurate approach to expression proteomics. Mol. Cell Proteomics.

[25] Berger SJ, Lee SW, Anderson GA, Pasa-Tolic L, Tolic N, Shen Y, Zhao R, Smith RD (2002). High-throughput global peptide proteomic analysis by combining stable isotope amino acid labeling and data-dependent multiplexed-MS/MS. Anal. Chem.

[26] Zhu H, Pan S, Gu S, Bradbury EM, Chen X (2002). Amino acid residue specific stable isotope labeling for quantitative proteomics. Rapid Commun. Mass Spectrom.

[27] Krijgsveld J, Ketting RF, Mahmoudi T, Johansen J, Artal-Sanz M, Verrijzer CP, Plasterk RH, Heck AJ (2003). Metabolic labeling of *C. elegans* and *D. melanogaster* for quantitative proteomics. Nat. Biotechnol.

[28] Wu CC, MacCoss MJ, Howell KE, Matthews DE, Yates JR III (2004). Metabolic labeling of mammalian organisms with stable isotopes for quantitative proteomic analysis. Anal. Chem.

[29] Ishihama Y, Sato T, Tabata T, Miyamoto N, Sagane K, Nagasu T, Oda Y (2005). Quantitative mouse brain proteomics using culture-derived isotope tags as internal standards. Nat. Biotechnol.

[30] Washburn MP, Ulaszek R, Deciu C, Schieltz DM, Yates JR III (2002). Analysis of quantitative proteomic data generated *via* multidimensional protein identification technology. Anal. Chem.

[31] Washburn MP, Koller A, Oshiro G, Ulaszek RR, Plouffe D, Deciu C, Winzeler E, Yates JR III (2003). Protein pathway and complex clustering of correlated mRNA and protein expression analyses in *Saccharomyces cerevisiae*. Proc. Natl. Acad. Sci. USA.

[32] Kolkman A, Olsthoorn MM, Heeremans CE, Heck AJ, Slijper M (2005). Comparative proteome analysis of *Saccharomyces cerevisiae* grown in chemostat cultures limited for glucose or ethanol. Mol. Cell Proteomics.

[33] Kolkman A, Daran-Lapujade P, Fullaondo A, Olsthoorn MM, Pronk JT, Slijper M, Heck AJ (2006). Proteome analysis of yeast response to various nutrient limitations. Mol. Syst. Biol.

[34] Ranish JA, Yi EC, Leslie DM, Purvine SO, Goodlett DR, Eng J, Aebersold R (2003). The study of macromolecular complexes by quantitative proteomics. Nat. Genet.

[35] Foster LJ, de Hoog CL, Mann M (2003). Unbiased quantitative proteomics of lipid rafts reveals high specificity for signaling factors. Proc. Natl. Acad. Sci. USA.

[36] Blagoev B, Kratchmarova I, Ong SE, Nielsen M, Foster LJ, Mann M (2003). A proteomics strategy to elucidate functional protein-protein interactions applied to EGF signaling. Nat. Biotechnol.

[37] Foster LJ, Rudich A, Talior I, Patel N, Huang X, Furtado LM, Bilan PJ, Mann M, Klip A (2006). Insulin-dependent interactions of proteins with GLUT4 revealed through stable isotope labeling by amino acids in cell culture (SILAC). J. Proteome Res.

[38] Pflieger D, Jünger MA, Müller M, Rinner O, Lee H, Gehrig PM, Gstaiger M, Aebersold R (2008). Quantitative proteomic analysis of protein complexes: concurrent identification of interactors and their state of phosphorylation. Mol. Cell Proteomics.

[39] Gingras AC, Gstaiger M, Raught B, Aebersold R (2007). Analysis of protein complexes using mass spectrometry. Nat. Rev. Mol. Cell Biol.

[40] Yang W, Steen H, Freeman MR (2008). Proteomic approaches to the analysis of multiprotein signaling complexes. Proteomics.

[41] Blagoev B, Ong SE, Kratchmarova I, Mann M (2004). Temporal analysis of phosphotyrosine-dependent signaling networks by quantitative proteomics. Nat. Biotechnol.

[42] Gruhler A, Olsen JV, Mohammed S, Mortensen P, Faergeman NJ, Mann M, Jensen ON (2005). Quantitative phosphoproteomics applied to the yeast pheromone signaling pathway. Mol. Cell Proteomics.

[43] Zhang Y, Wolf-Yadlin A, Ross PL, Pappin DJ, Rush J, Lauffenburger DA, White FM (2005). Time-resolved mass spectrometry of tyrosine phosphorylation sites in the epidermal growth factor receptor signaling network reveals dynamic modules. Mol. Cell Proteomics.

[44] Olsen JV, Blagoev B, Gnad F, Macek B, Kumar C, Mortensen P, Mann M (2006). Global, *in vivo*, and site-specific phosphorylation dynamics in signaling networks. Cell.

[45] Li X, Gerber SA, Rudner AD, Beausoleil SA, Haas W, Villén J, Elias JE, Gygi SP (2007). Large-scale phosphorylation analysis of alpha-factor-arrested *Saccharomyces cerevisiae*. J. Proteome Res.

[46] Desiderio DM, Kai M (1983). Preparation of stable isotope-incorporated peptide internal standards for field desorption mass spectrometry quantification of peptides in biologic tissue. Biomed. Mass Spectrom.

[47] Kusmierz JJ, Sumrada R, Desiderio DM (1990). Fast atom bombardment mass spectrometric quantitative analysis of methionine-enkephalin in human pituitary tissues. Anal. Chem.

[48] Barr JR, Maggio VL, Patterson DG Jr, Cooper GR, Henderson LO, Turner WE, Smith SJ, Hannon WH, Needham LL, Sampson EJ (1996). Isotope dilution--mass spectrometric quantification of specific proteins: model application with apolipo-protein A-I. Clin. Chem.

[49] Barnidge DR, Dratz EA, Martin T, Bonilla LE, Moran LB, Lindall A (2003). Absolute quantification of the G protein-coupled receptor rhodopsin by LC/MS/MS using proteolysis product peptides and synthetic peptide standards. Anal. Chem.

[50] Gerber SA, Rush J, Stemman O, Kirschner MW, Gygi SP (2003). Absolute quantification of proteins and phosphoproteins from cell lysates by tandem MS. Proc. Natl. Acad. Sci. USA.

[51] Kuhn E, Wu J, Karl J, Liao H, Zolg W, Guild B (2004). Quantification of C-reactive protein in the serum of patients with rheumatoid arthritis using multiple reaction monitoring mass spectrometry and ^13^C-labeled peptide standards. Proteomics.

[52] Zhang F, Bartels MJ, Stott WT (2004). Quantitation of human glutathione S-transferases in complex matrices by liquid chromatography/tandem mass spectrometry with signature peptides. Rapid Commun. Mass Spectrom.

[53] Hochleitner EO, Kastner B, Fröhlich T, Schmidt A, Lührmann R, Arnold G, Lottspeich F (2005). Protein stoichiometry of a multiprotein complex, the human spliceosomal U1 small nuclear ribonucleoprotein: absolute quantification using isotope-coded tags and mass spectrometry. J. Biol. Chem.

[54] Pan S, Zhang H, Rush J, Eng J, Zhang N, Patterson D, Comb MJ, Aebersold R (2005). High throughput proteome screening for biomarker detection. Mol. Cell Proteomics.

[55] Cheng D, Hoogenraad CC, Rush J, Ramm E, Schlager MA, Duong DM, Xu P, Wijayawardana SR, Hanfelt J, Nakagawa T, Sheng M, Peng J (2006). Relative and absolute quantification of postsynaptic density proteome isolated from rat forebrain and cerebellum. Mol. Cell Proteomics.

[56] Stemmann O, Zou H, Gerber SA, Gygi SP, Kirschner MW (2001). Dual inhibition of sister chromatid separation at metaphase. Cell.

[57] Mayya V, Rezual K, Wu L, Fong MB, Han DK (2006). Absolute quantification of multisite phosphorylation by selective reaction monitoring mass spectrometry: determination of inhibitory phosphorylation status of cyclin-dependent kinases. Mol. Cell Proteomics.

[58] Kirkpatrick DS, Gerber SA, Gygi SP (2005). The absolute quantification strategy a general procedure for the quantification of proteins and post-translational modifications. Methods.

[59] Kirkpatrick DS, Hathaway NA, Hanna J, Elsasser S, Rush J, Finley D, King RW, Gygi SP (2006). Quantitative analysis of *in vitro* ubiquitinated cyclin B1 reveals complex chain topology. Nat. Cell Biol.

[60] Appels NM, Rosing H, Stephens TC, Hughes A, Schellens JH, Beijnen JH (2006). Absolute quantification of farnesylated Ras levels in complex samples using liquid chromatography fractionation combined with tryptic digestion and electrospray tandem mass spectrometry. Anal. Biochem.

[61] Havlis J, Shevchenko A (2004). Absolute quantification of proteins in solutions and in polyacrylamide gels by mass spectrometry. Anal. Chem.

[62] Kippen AD, Cerini F, Vadas L, Stöcklin R, Vu L, Offord RE, Rose K (1997). Development of an isotope dilution assay for precise determination of insulin, C-peptide, and proinsulin levels in non-diabetic and type II diabetic individuals with comparison to immunoassay. J. Biol. Chem.

[63] Peng J, Kim MJ, Cheng D, Duong DM, Gygi SP, Sheng M (2004). Semiquantitative proteomic analysis of rat forebrain postsynaptic density fractions by mass spectrometry. J. Biol. Chem.

[64] Janecki DJ, Bemis KG, Tegeler TJ, Sanghani PC, Zhai L, Hurley TD, Bosron WF, Wang M (2007). A multiple reaction monitoring method for absolute quantification of the human liver alcohol dehydrogenase ADH1C1 isoenzyme. Anal. Biochem.

[65] Brun V, Dupuis A, Adrait A, Marcellin M, Thomas D, Court M, Vandenesch F, Garin J (2007). Isotope-labeled protein standards: toward absolute quantitative proteomics. Mol. Cell Proteomics.

[66] Beynon RJ, Doherty MK, Pratt JM, Gaskell SJ (2005). Multiplexed absolute quantification in proteomics using artificial QCAT proteins of concatenated signature peptides. Nat. Methods.

[67] Kito K, Ota K, Fujita T, Ito T (2007). A synthetic protein approach toward accurate mass spectrometric quantification of component stoichiometry of multiprotein complexes. J. Proteome Res.

[68] Rivers J, Simpson DM, Robertson DH, Gaskell SJ, Beynon RJ (2007). Absolute multiplexed quantitative analysis of protein expression during muscle development using QconCAT. Mol. Cell Proteomics.

[69] Anderson L, Hunter CL (2006). Quantitative mass spectrometric multiple reaction monitoring assays for major plasma proteins. Mol. Cell Proteomics.

[70] Yen CY, Russell S, Mendoza AM, Meyer-Arendt K, Sun S, Cios KJ, Ahn NG, Resing KA (2006). Improving sensitivity in shotgun proteomics using a peptide-centric database with reduced complexity: protease cleavage and SCX elution rules from data mining of MS/MS spectra. Anal. Chem.

[71] Siepen JA, Keevil EJ, Knight D, Hubbard SJ (2007). Prediction of missed cleavage sites in tryptic peptides aids protein identification in proteomics. J. Proteome Res.

[72] Nanavati D, Gucek M, Milne JL, Subramaniam S, Markey SP (2008). Stoichiometry and absolute quantification of proteins with mass spectrometry using fluorescent and isotope-labeled concatenated peptide standards. Mol. Cell Proteomics.

[73] Mirzaei H, McBee J, Watts J, Aebersold R (2007). Comparative evaluation of current peptide production platforms used in absolute quantification in proteomics. Mol. Cell Proteomics.

[74] Old WM, Meyer-Arendt K, Aveline-Wolf L, Pierce KG, Mendoza A, Sevinsky JR, Resing KA, Ahn NG (2005). Comparison of label-free methods for quantifying human proteins by shotgun proteomics. Mol. Cell Proteomics.

[75] Wang G, Wu WW, Zeng W, Chou CL, Shen RF (2006). Label-free protein quantification using LC-coupled ion trap or FT mass spectrometry: Reproducibility, linearity, and application with complex proteomes. J. Proteome Res.

[76] Andersen JS, Wilkinson CJ, Mayor T, Mortensen P, Nigg EA, Mann M (2003). Proteomic characterization of the human centrosome by protein correlation profiling. Nature.

[77] Rinner O, Mueller LN, Hubálek M, Müller M, Gstaiger M, Aebersold R (2007). An integrated mass spectrometric and computational framework for the analysis of protein interaction networks. Nat. Biotechnol.

[78] Kim YJ, Zhan P, Field B, Ruben SM, He T (2007). Reproducibility assessment of relative quantitation strategies for LC-MS based proteomics. Anal. Chem.

[79] Bondarenko PV, Chelius D, Shaler TA (2002). Identification and relative quantitation of protein mixtures by enzymatic digestion followed by capillary reversed-phase liquid chromatography-tandem mass spectrometry. Anal. Chem.

[80] Wang W, Zhou H, Lin H, Roy S, Shaler TA, Hill LR, Norton S, Kumar P, Anderle M, Becker CH (2003). Quantification of proteins and metabolites by mass spectrometry without isotopic labeling or spiked standards. Anal. Chem.

[81] Tabata T, Sato T, Kuromitsu J, Oda Y (2007). Pseudo internal standard approach for label-free quantitative proteomics. Anal. Chem.

[82] Jaitly N, Monroe ME, Petyuk VA, Clauss TR, Adkins JN, Smith RD (2006). Robust algorithm for alignment of liquid chromatography-mass spectrometry analyses in an accurate mass and time tag data analysis pipeline. Anal. Chem.

[83] Pang JX, Ginanni N, Dongre AR, Hefta SA, Opitek GJ (2002). Biomarker discovery in urine by proteomics. J. Proteome Res.

[84] Gao J, Opiteck GJ, Friedrichs MS, Dongre AR, Hefta SA (2003). Changes in the protein expression of yeast as a function of carbon source. J. Proteome Res.

[85] Zybailov B, Coleman MK, Florens L, Washburn MP (2005). Correlation of relative abundance ratios derived from peptide ion chromatograms and spectrum counting for quantitative proteomic analysis using stable isotope labeling. Anal. Chem.

[86] Asara JM, Christofk HR, Freimark LM, Cantley LC (2008). A label-free quantification method by MS/MS TIC compared to SILAC and spectral counting in a proteomics screen. Proteomics.

[87] Silva JC, Gorenstein MV, Li GZ, Vissers JP, Geromanos SJ (2006). Absolute quantification of proteins by LCMS^E^: a virtue of parallel MS acquisition. Mol. Cell Proteomics.

[88] Blondeau F, Ritter B, Allaire PD, Wasiak S, Girard M, Hussain NK, Angers A, Legendre-Guillemin V, Roy L, Boismenu D, Kearney RE, Bell AW, Bergeron JJ, McPherson PS (2004). Tandem MS analysis of brain clathrin-coated vesicles reveals their critical involvement in synaptic vesicle recycling. Proc. Natl. Acad. Sci. USA.

[89] Powell DW, Weaver CM, Jennings JL, McAfee KJ, He Y, Weil PA, Link AJ (2004). Cluster analysis of mass spectrometry data reveals a novel component of SAGA. Mol. Cell Biol.

[90] Zybailov B, Mosley AL, Sardiu ME, Coleman MK, Florens L, Washburn MP (2006). Statistical analysis of membrane proteome expression changes in *Saccharomyces cerevisiae*. J. Proteome Res.

[91] Paoletti AC, Parmely TJ, Tomomori-Sato C, Sato S, Zhu D, Conaway RC, Conaway JW, Florens L, Washburn MP (2006). Quantitative proteomic analysis of distinct mammalian Mediator complexes using normalized spectral abundance factors. Proc. Natl. Acad. Sci. USA.

[92] Rappsilber J, Ryder U, Lamond AI, Mann M (2002). Large-scale proteomic analysis of the human spliceosome. Genome Res.

[93] Ishihama Y, Oda Y, Tabata T, Sato T, Nagasu T, Rappsilber J, Mann M (2005). Exponentially modified protein abundance index (emPAI) for estimation of absolute protein amount in proteomics by the number of sequenced peptides per protein. Mol. Cell Proteomics.

[94] Tang H, Arnold RJ, Alves P, Xun Z, Clemmer DE, Novotny MV, Reilly JP, Radivojac P (2006). A computational approach toward label-free protein quantification using predicted peptide detectability. Bioinformatics.

[95] Lu P, Vogel C, Wang R, Yao X, Marcotte EM (2007). Absolute protein expression profiling estimates the relative contributions of transcriptional and translational regulation. Nat. Biotechnol.

[96] Nesvizhskii AI, Keller A, Kolker E, Aebersold R (2003). A statistical model for identifying proteins by tandem mass spectrometry. Anal. Chem.

[97] Mallick P, Schirle M, Chen SS, Flory MR, Lee H, Martin D, Ranish J, Raught B, Schmitt R, Werner T, Kuster B, Aebersold R (2007). Computational prediction of proteotypic peptides for quantitative proteomics. Nat. Biotechnol.

[98] Kuster B, Schirle M, Mallick P, Aebersold R (2005). Scoring proteomes with proteotypic peptide probes. Nat. Rev. Mol. Cell Biol.

[99] Craig R, Cortens JP, Beavis RC (2005). The use of proteotypic peptide libraries for protein identification. Rapid Commun. Mass Spectrom.

[100] Sanders WS, Bridges SM, McCarthy FM, Nanduri B, Burgess SC (2007). Prediction of peptides observable by mass spectrometry applied at the experimental set level. BMC Bioinformatics.

[101] Wei J, Sun J, Yu W, Jones A, Oeller P, Keller M, Woodnutt G, Short JM (2005). Global proteome discovery using an online three-dimensional LC-MS/MS. J. Proteome Res.

[102] de Godoy LM, Olsen JV, de Souza GA, Li G, Mortensen P, Mann M (2006). Status of complete proteome analysis by mass spectrometry: SILAC labeled yeast as a model system. Genome Biol.

[103] Ghaemmaghami S, Huh WK, Bower K, Howson RW, Belle A, Dephoure N, O'Shea EK, Weissman JS (2003). Global analysis of protein expression in yeast. Nature.

[104] Brunner E, Ahrens CH, Mohanty S, Baetschmann H, Loevenich S, Potthast F, Deutsch EW, Panse C, de Lichtenberg U, Rinner O, Lee H, Pedrioli PG, Malmstrom J, Koehler K, Schrimpf S, Krijgsveld J, Kregenow F, Heck AJ, Hafen E, Schlapbach R, Aebersold R (2007). A high-quality catalog of the *Drosophila melanogaster* proteome. Nat. Biotechnol.

[105] Elias JE, Haas W, Faherty BK, Gygi SP (2005). Comparative evaluation of mass spectrometry platforms used in large-scale proteomics investigations. Nat. Methods.

[106] Krogan NJ, Cagney G, Yu H, Zhong G, Guo X, Ignatchenko A, Li J, Pu S, Datta N, Tikuisis AP, Punna T, Peregrín-Alvarez JM, Shales M, Zhang X, Davey M, Robinson MD, Paccanaro A, Bray JE, Sheung A, Beattie B, Richards DP, Canadien V, Lalev A, Mena F, Wong P, Starostine A, Canete MM, Vlasblom J, Wu S, Orsi C, Collins SR, Chandran S, Haw R, Rilstone JJ, Gandi K, Thompson NJ, Musso G, St Onge P, Ghanny S, Lam MH, Butland G, Altaf-Ul AM, Kanaya S, Shilatifard A, O'Shea E, Weissman JS, Ingles CJ, Hughes TR, Parkinson J, Gerstein M, Wodak SJ, Emili A, Greenblatt JF (2006). Global landscape of protein complexes in the yeast *Saccharomyces cerevisiae*. Nature.

[107] Keshishian H, Addona T, Burgess M, Kuhn E, Carr SA (2007). Quantitative, multiplexed assays for low abundance proteins in plasma by targeted mass spectrometry and stable isotope dilution. Mol. Cell Proteomics.

[108] Scigelova M, Makarov A (2006). Orbitrap mass analyzer - overview and applications in proteomics. Proteomics.

[109] Hanke S, Besir H, Oesterhelt D, Mann M (2008). Absolute SILAC for accurate quantitation of proteins in complex mixtures down to the attomole level. J. Proteome Res.

